# Surgeons’ participation in the development of collaboration and management competencies in undergraduate medical education

**DOI:** 10.1371/journal.pone.0233400

**Published:** 2020-06-05

**Authors:** Miriam Rothdiener, Jan Griewatz, Adrian Meder, Alessandro Dall’Acqua, Udo Obertacke, Andreas Kirschniak, Katrin Borucki, Sarah Koenig, Miriam Ruesseler, Sandra Steffens, Bernhard Steinweg, Maria Lammerding-Koeppel

**Affiliations:** 1 Competence Centre for University Teaching in Medicine, Baden-Wuerttemberg, Faculty of Medicine, University of Tuebingen, Tuebingen, Germany; 2 Department of Trauma and Reconstructive Surgery, Berufsgenossenschaftliche Unfallklinik Tuebingen, University of Tuebingen, Tuebingen, Germany; 3 Competence Centre for Evaluation of Teaching in Medicine, Baden-Wuerttemberg, Faculty of Medicine, University of Freiburg, Freiburg, Germany; 4 Orthopaedic and Trauma Surgery Center, University Medicine Mannheim, Medical Faculty Mannheim of the University of Heidelberg, Mannheim, Germany; 5 Competence Centre of Final Year, Medical Faculty Mannheim, University of Heidelberg, Mannheim, Germany; 6 Department of General, Visceral and Transplant Surgery, University Hospital Tuebingen, Tuebingen, Germany; 7 Institute for Clinical Chemistry and Pathobiochemistry, University of Magdeburg, Magdeburg, Germany; 8 Insitute for Medical Teaching and Medical Education Research, University Hospital Wuerzburg, Wuerzburg, Germany; 9 Department of Trauma, Hand and Reconstructive Surgery, University Hospital Frankfurt, Frankfurt, Germany; 10 Dean’s Office for Medical Education, Hannover Medical School, Hannover, Germany; 11 Department of Paediatric Cardiology, University of Bonn, Bonn, Germany; University Hospital Zurich, SWITZERLAND

## Abstract

The teaching of professional roles in medical education is an interdisciplinary concern. However, surgeons require specific standards of professionalism for certain context-based situations. In addition to communication, studies require collaboration, leadership, error-/conflict-management, patient-safety and decision-making as essential competencies for surgeons. Standards for corresponding competencies are defined in special chapters of the German National Competency-based Learning Objectives for Undergraduate Medical Education (NKLM; chapter 8, 10). The current study asks whether these chapters are adequately taught in surgical curricula. Eight German faculties contributed to analysing mapping data considering surgical courses of undergraduate programs. All faculties used the MER*lin* mapping platform and agreed on procedures for data collection and processing. Sub-competency and objective coverage, as well as the achievement of the competency level were mapped. Overall counts of explicit citations were used for analysis. Collaboration within the medical team is a strongly represented topic. In contrast, interprofessional cooperation, particularly in healthcare sector issues is less represented. Patient safety and dealing with errors and complications is most emphasized for the Manager/Leader, while time management, career planning and leadership are not addressed. Overall, the involvement of surgery in teaching the competencies of the Collaborator and Manager/Leader is currently low. However, there are indications of a curricular development towards explicit teaching of these roles in surgery. Moreover, implicitly taught roles are numerous, which indicates a beginning awareness of professional roles.

## Introduction

The teaching of professional roles in medical education is an interdisciplinary issue, as all relevant and typical facets of tasks in physicians’ daily practice are concerned. However, insufficient implementation of professional roles in medical education can lead to problems and errors in practice. Especially in the operating room, interpersonal conflicts remain a significant problem [1]. The operating room is also described as one of the main sources of medical error [2, 3], whereas a substantial part of these adverse events are avoidable [3]. Symptomatically, a large proportion of surgical errors are caused by non-technical errors of competency [4]. Human factors are one example [5], another is poor communication between the members of a team [6]. Studies investigating the need for psychosocial skills in surgery point to collaboration, leadership, patient safety, including mainly error- and conflict management, as well as decision-making as essential needs in addition to communication [7–12]. Interestingly, in the study by Arora et al., no surgeon stated that they had achieved competence in every professional role, but there is an improvement over time of practice. However, the competencies of Manager/Leader (in the following abbreviated as Manager) and Collaborator are not described in such a way that they are fully mastered by the respondents in this study [8]. The study is supported by further publications: Cope et al. found that essential surgeons’ attitudes such as (self-) management, teamwork and leadership are learned at a post-graduate level [13]. Frank et al. also described only the post-graduate teaching of collaboration, communication, management and advocacy [14]. Based on these findings and in view of the increasing public concern about surgical competencies [7], it is fundamental to develop a defined explicit basic level of competency early in surgical education. Moreover, surgeons require specific standards for particular context-based situations, as professional skills cannot be segregated from technical skills. Amongst others, cognitive skills such as problem anticipation and decision-making are necessary to improve technical performance [15].

In Germany, collaboration and management competencies are explicitly described in chapters 8 (Collaborator) and 10 (Manager) of the National Competency-based Learning Objectives for Undergraduate Medical Education (NKLM) [16], a comprehensive framework, including scientific and research-related standards. Professional roles of the NKLM are based on the CanMEDs [17]. The German Society for Medical Education (GMA) and the Association of Medical Faculties in Germany (MFT) developed the NKLM, revised by the Association of the Scientific Medical Societies in Germany (AWMF) [18]. Competency levels are defined at certain milestones in the catalogue. Professional roles are characterized by communication and interaction with different groups and by working in intersecting settings.

The Collaborator describes the work of physicians in an interprofessional health care team. The central purpose of this role is to prevent, negotiate and resolve interpersonal and interprofessional conflicts [19–21]. The role as Manager describes important management and leadership competencies that are necessary in dealing with complex situations. In clinical care, health economics, healthcare strategy, personnel and process management have to be considered and balanced [22]. This includes initiating and facilitating shared, collaborative decision-making at the level of patients and healthcare system management [22, 23].

In order to avoid medical error situations due to a lack of competencies in collaboration and management and to provide opportunities to acquire these competencies at an early stage, it is essential to include corresponding learning settings in undergraduate education. In addition, the way in which theoretically defined competencies are taught in educational practice is of high relevance for sustainable learning [24]. It is also important to consider specific surgical contexts rather than relying on interdisciplinary teaching of professional roles. A clear positioning of a single discipline, embedded in the curriculum of a faculty is desirable. The aim of the present study was therefore to investigate the role of collaborative working and efficient management in the curricula of surgical education at eight representative German medical faculties. We hypothesize that essential team and management competencies are not adequately taught in surgery.

## Methods

### Procedure

The surgery courses of the undergraduate courses of study at eight German medical faculties (Tuebingen, Freiburg, Mannheim, Bonn, Frankfurt, Hannover, Magdeburg and Wuerzburg) were mapped from the perspective of the teaching staff, using prescribed national standards (NKLM). The term “course” was defined as the basic unit of representation, which can stand for courses of varying lengths. All obligatory curricular courses which belong to the classic surgical disciplines were included, in the case of trauma surgery and neurosurgery, content from orthopaedics and neurology were also included.

The project was managed by the Competence Centre of University Teaching in Medicine Baden-Wuerttemberg in Tuebingen (CCMD). To ensure consistent process and data quality, all faculties used the web-based mapping platform MER*lin* [25] as a common tool and followed a coordinated approach, supported by instruction and individual advice from the CCMD staff. Each faculty entered its curriculum data into a protected data space of the MER*lin* platform. The mapping tool, the procedures as well as the methods of data collection and processing were described in detail earlier: Mapping tool and associated procedures are exemplified from the application perspective. The mapper is guided by the user interface displaying the mapping structure. The mapping structure follows the NKLM hierarchy of Chapters (Chapt.), Competencies (C), sub-competencies (SC) and underlying objectives (O), wording is in analogy to the original catalogue. Course characterization is conducted on the sub-competency level, related learning objectives give more details and are to be ticked off if taught [25]. The mapping at sub-competency level was carried out by selecting pre-defined menu options: (a) the highest level of competency achieved (CL); (b) transparency in teaching (“explicit” means written in a study-guide, module manual or other material); (c) the extent of completeness of the sub-competency as automatically calculated from the underlying learning objectives. Mapping citations refer to any objective taught in a course. The teaching of an objective once or several times in a course corresponds to one citation. Competencies, sub-competencies and underlying objective of each professional role were identified by the code numbers of NKLM chapter. (Short descriptions of competencies and objectives are given in the figures, for complete versions of the unofficial translation see S1 Appendix).

Local datasets of the medical faculties were anonymized by serial numbers in random order.

In order to ensure the validity of the content, the mapping was carried out by up to seven individuals from the surgical discipline of each site, often preceptors with expertise of courses or senior teachers with educational background, who coordinate and/or supervise the courses of the department. Plausibility checks were carried out by local authorized representatives and/or dean’s office. The global administrator of CCMD performed regular consistency checks.

### Analysis

Eight medical faculties contributed to the current analysis of the mapping data, focusing on the explicit curricular representation of two professional roles in surgery: Collaborator and Manager (NKLM Chapt. 8 and 10). Explicit and implicit sub-competencies and objectives and competency levels achieved were recorded, whereby only explicit items were considered for the analysis. For the statistical evaluation of the data with Excel (Microsoft Office Package, 2010), descriptive statistics with percentages, frequencies, mean values, minimum and maximum were used (Tables 1 and 2).

**Table 1:** Relative frequencies and statistical data of Chapt. 8 objectives.

Objective	MF1	MF2	MF3	MF4	MF5	MF6	MF7	MF8	Mdn	Min-Max	Mean	SD
8.1.1.1	3.7	1.2	1.2	0	0	0	2.4	0	0.6	0–3.7	**1.1**	1.4
8.1.1.2	2.4	1.2	1.2	0	0	0	3.7	0	0.6	0–3.7	**1.1**	1.4
8.1.1.3	4.9	0	1.2	0	0	0	3.7	0	0	0–4.9	**1.2**	2.0
8.1.2.1	1.2	1.2	0	0	0	0	2.4	0	0	0–2.4	0.6	0.9
8.1.2.2	1.2	0	0	0	0	0	1.2	0	0	0–1.2	0.3	0.6
8.1.2.3	1.2	0	0	0	0	0	1.2	0	0	0–1.2	0.3	0.6
8.2.1.1	6.1	0	0	0	0	0	2.4	0	0	0–6.1	**1.1**	2.2
8.2.1.2	6.1	0	0	1.2	0	0	1.2	0	0	0–6.1	**1.1**	2.1
8.2.1.3	6.1	0	0	0	0	0	0	0	0	0–6.1	0.8	2.1
8.2.2.1	2.4	0	0	0	0	0	3.7	0	0	0–3.7	0.8	1.4
8.2.2.2	2.4	0	0	0	0	0	2.4	0	0	0–2.4	0.6	1.1
8.2.2.3	1.2	0	0	0	0	0	0	0	0	0–1.2	0.2	0.4
8.2.3.1	1.2	0	0	0	0	0	1.2	0	0	0–1.2	0.3	0.6
8.2.3.2	1.2	0	0	0	0	0	1.2	0	0	0–1.2	0.3	0.6
8.3.1.1	0	0	0	0	0	1.2	1.2	0	0	0–1.2	0.3	0.6
8.3.1.2	0	1.2	1.2	1.2	0	2.4	1.2	0	1.2	0–2.4	0.9	0.9
8.3.2.1	0	0	1.2	1.2	0	1.2	1.2	0	0.6	0–1.2	0.6	0.7
8.3.3.1	1.2	0	0	0	0	0	1.2	0	0	0–1.2	0.3	0.6
8.3.3.2	2.4	0	0	1.2	0	0	1.2	0	0	0–2.4	0.6	0.9
8.4.1.1	0	0	0	0	0	0	1.2	0	0	0–1.2	0.2	0.4
8.4.1.2	0	0	0	0	0	0	0	0	0	0	0	0
8.4.1.3	0	0	0	0	0	0	0	0	0	0	0	0
8.4.2.1	0	0	0	0	0	0	0	0	0	0	0	0
8.4.2.2	0	0	0	0	0	0	0	0	0	0	0	0

**MF** Medical Faculty, **Mdn** Median, **Min** Minimum, **Max** Maximum, **SD** Standard Deviation

**Table 2:** Relative frequencies and statistical data of Chapt. 10 objectives.

Objective	MF1	MF2	MF3	MF4	MF5	MF6	MF7	MF8	Mdn	Min-Max	Mean	SD
10.1.1.1	1.9	0	0	0	0	0	1.9	0	0	0–1.9	0.5	0.9
10.1.1.2	0	0	0	0	0	0	0	0	0	0	0	0
10.1.1.3	0	0	0	0	0	0	0	0	0	0	0	0
10.2.1.1	1.9	0	0	0	0	0	1.9	1.9	0	0–1.9	0.7	1.0
10.3.1.1	0	0	0	0	0	0	0	0	0	0	0	0
10.3.1.2	0	0	0	0	0	0	0	0	0	0	0	0
10.3.1.3	0	0	0	0	0	0	0	0	0	0	0	0
10.3.1.4	0	0	0	1.9	0	0	0	0	0	0–1.9	0.2	0.7
10.3.2.1	0	0	0	0	0	0	1.9	0	0	0–1.9	0.2	0.7
10.3.2.2	0	0	0	0	0	0	0	0	0	0	0	0
10.4.1.1	0	0	0	0	0	0	0	0	0	0	0	0
10.4.1.2	0	0	0	0	0	0	0	0	0	0	0	0
10.4.1.3	0	0	0	0	0	0	0	0	0	0	0	0
10.4.2.1	0	0	0	0	0	0	0	0	0	0	0	0
10.5.1.1	0	0	1.9	0	0	0	1.9	0	0	0–1.9	0.5	0.9
10.6.1.1	0	0	0	0	0	1.9	5.7	1.9	0	0–5.7	1.2	2,0
10.6.1.2	3.8	0	0	1,9	0	3.8	7.5	1.9	1.9	0–7.5	**2.4**	2.6
10.6.1.3	1.9	0	0	0	0	1.9	1.9	1.9	0.9	0–1.9	0.9	1.0
10.6.1.4	3.8	0	0	1.9	0	1.9	3.8	1.9	1.9	0–3.8	**1.7**	1.6
10.6.1.5	0	0	0	0	0	0	0	1.9	0	0–1.9	0.2	0.7
10.6.2.1	0	0	0	0	0	0	0	1.9	0	0–1.9	0.2	0.7
10.6.2.2	0	0	0	0	0	0	0	1.9	0	0–1.9	0.2	0.7
10.6.3.1	1.9	0	0	1.9	0	0	3.8	0	0	0–3.8	0.9	1.4
10.6.3.2	1.9	0	0	0	0	0	0	0	0	0–1.9	0.2	0.7
10.6.3.3	0	0	0	0	0	1.9	0	0	0	0–1.9	0.2	0.7
10.7.1.1	0	0	0	0	0	0	1.9	0	0	0–1.9	0.2	0.7
10.7.1.2	0	1.9	0	0	0	0	1.9	0	0	0–1.9	0.5	0.9
10.7.1.3	0	1.9	0	0	0	0	0	0	0	0–1.9	0.2	0.7
10.7.1.4	0	0	0	1.9	0	0	1.9	0	0	0–1.9	0.5	0.9
10.7.1.5	0	0	0	0	0	0	0	0	0	0	0	0
10.8.1.1	0	0	0	0	0	0	0	0	0	0	0	0
10.9.1.1	0	0	0	0	0	0	1.9	0	0	0–1.9	0.2	0.7
10.9.2.1	0	0	0	0	0	0	0	0	0	0	0	0
10.10.1.1	0	0	0	0	0	1.9	0	0	0	0–1.9	0.2	0.7
10.10.1.2	0	0	0	0	0	0	0	0	0	0	0	0
10.10.2.1	0	0	0	0	0	0	1.9	0	0	0–1.9	0.2	0.7
10.10.2.2	0	0	0	0	0	0	0	0	0	0	0	0

**MF** Medical Faculty, **Mdn** Median, **Min** Minimum, **Max** Maximum, **SD** Standard Deviation

#### Relative frequency and weighting

The values of the faculties were obtained by calculating the frequency and weighting of the sub-competencies and objectives of the individual locations in relation to the total equivalents of the corresponding chapters. The procedure did not aim to compare the single programmes, but rather to give a comprehensive representation of the sub-competencies and objectives in surgery and to determine the contents of these chapters that received the most attention. Strengths and weaknesses in team and management competencies in surgical education can thus be visualized, presuming that the more courses represent an objective (mapping citations), the higher the curricular emphasis. To calculate the relative curricular frequency of a sub-competency at a medical faculty (MFx), the number of citations (n_Cit_) was put in the context of the total number of citations for the corresponding chapter at all sites (N_Cit_). The considerations led to the following formula:

$$Relative\;Frequency\;(SC;\;MFx)=\frac{nCit\;(SC;MFx)}{NCit\;(\Sigma MF;Chapt)}*100$$

The formula is also correct for overall relative frequency of objectives:

$$Relative\;Frequency\;(O;\;MFx)=\frac{nCit\;(O;MFx)}{NCit\;(\Sigma MF;Chapt)}*100$$

**Fig 1:**
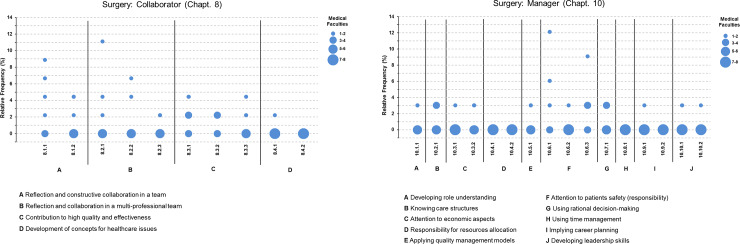
Relative teaching frequency of Sub-Competencies (SC) in surgery. **A** Collaborator; **B** Manager. Each faculty’s frequency for a single SC was put in context of total numbers of citations for the chapter at all faculties. Circle diameter encodes for number of faculties teaching a SC explicitly. See appendix for detailed description of SC.

In order to visualize the representation of sub-competencies, a modified scatter diagram with data of the eight programmes was created for each chapter, depicting the relative frequency for each individual programme (Fig 1). To provide a more detailed view, relative frequencies were also calculated for every objective and location (Tables 1 and 2).

**Fig 2:**
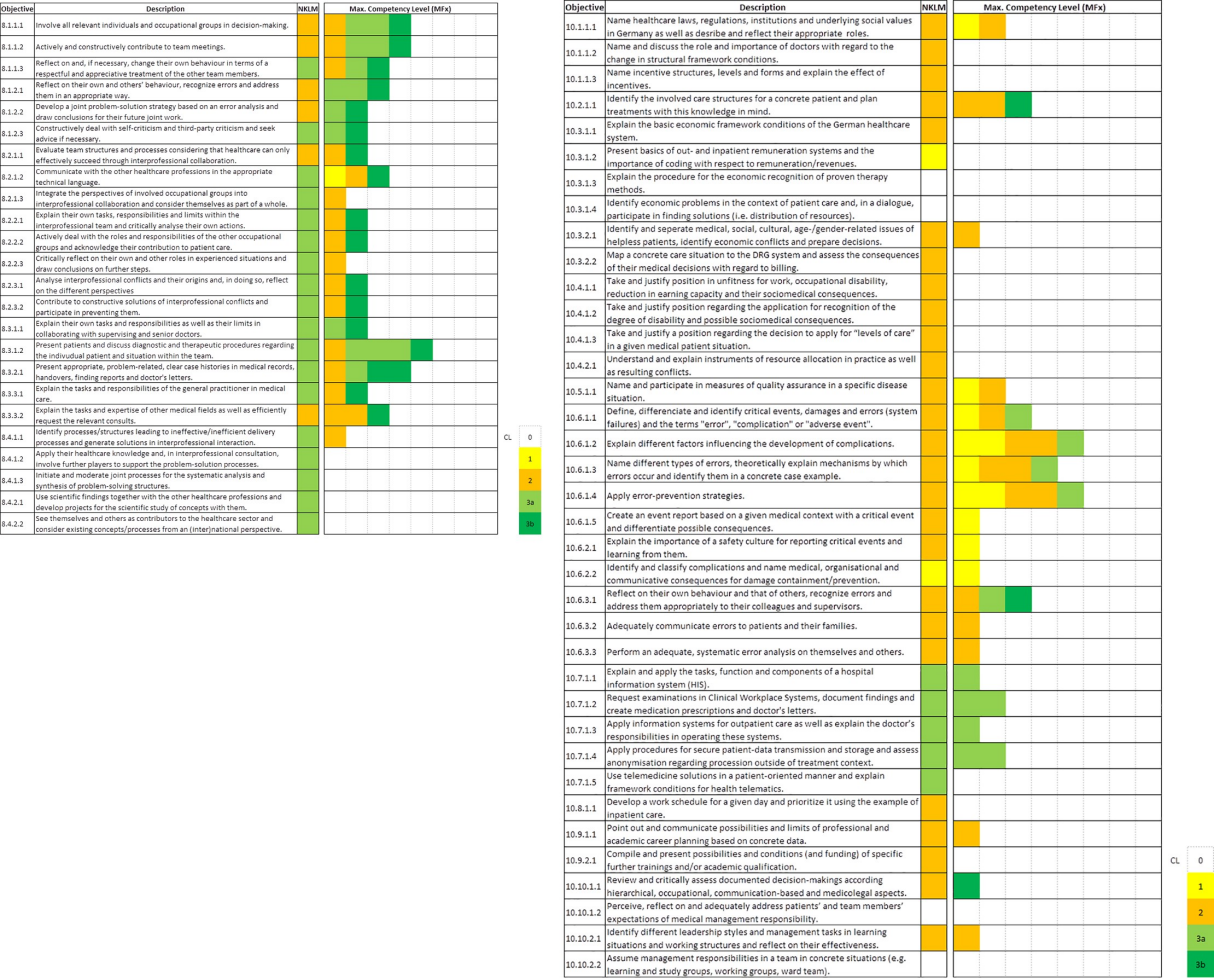
Competency Levels (CL) of learning objectives (O) in surgery across sites. **A** Collaborator; **B** Manager. The eight participating sites are represented by a box each. Each faculty’s maximum CL opposed to given standard (NKLM). CL: no standard (0, white) knowledge/understanding/basic skills (1, yellow), applied knowledge and skills in training (2, orange), competency in practice, supervised (3a, light green) and independent (3b, dark green). Columns do not represent specific faculties but reveal the cross-site profile.

For analysing the actual quality of teaching, the highest competency levels of the learning objectives achieved after five years of studies (milestone: entry in the practical year) were shown in an overview in Fig 2. They were compared to the NKLM requirements as given reference, separately for each location. The study included four levels of competency: knowledge/understanding/basic skills (1), applied knowledge and skills in training (2), competency in practice, supervised (3a) and independent (3b). The following criteria were applied to highlight significant objectives: number of faculties and competency level achieved. Due to the lower curricular occurrence of chapter 10 contents, criteria were adopted: Objectives were graded as prominent in chapter 8 if they meet criteria “citations from at least four sites and highest competency level of 4”; and in chapter 10 either “citations from at least four sites and highest competency level of 3”, or “citations from at least one site and highest competency level of 4”.

**Fig 3:**
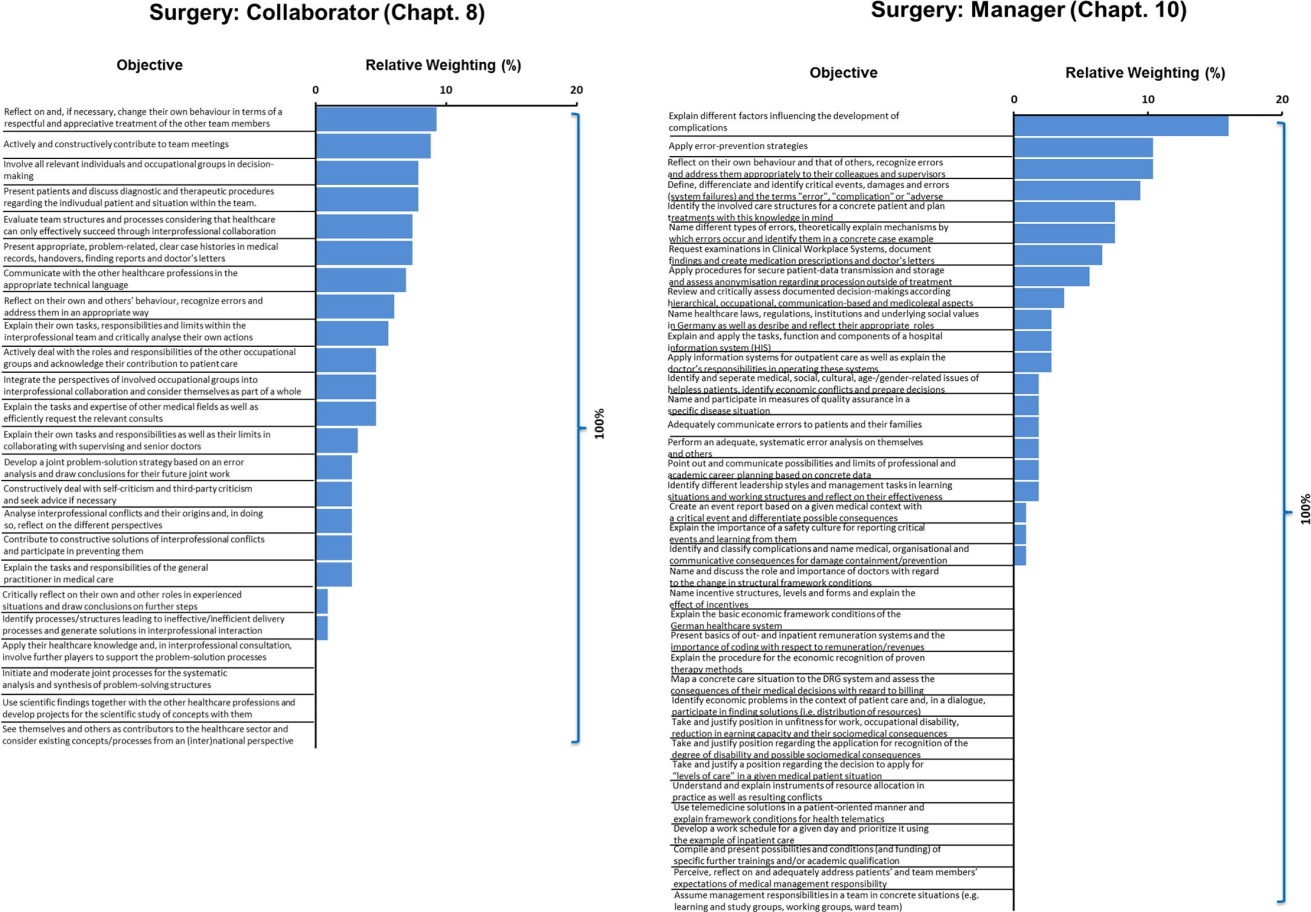
Relative weighting of learning objectives (O) in surgery. **A** Collaborator; **B** Manager. Weighted values were calculated based on absolute values of mapping citations multiplied by absolute competency levels. Each faculty’s weighted values were put in context of total weighted values for the chapters at all faculties. For ranking of O, relative weightings of all faculties were summed up.

In order to visualize the degree of implementation of objectives in the surgical curricula, we combined their frequencies and competency levels (Fig 3). In a first step, weighted values of mapping citations were determined. They were calculated based on absolute values of mapping citations multiplied by absolute competency levels. Therefore, the levels 1, 2, 3a and 3b were coded as factors 1, 2, 3 and 4 respectively.

$$Weighting\;\left(O;\;MFx\right)=\left(n_{Cit\;(O;\;MFx)}xCL_{Cit\;(O;\;MFx)}\right)$$

To achieve comprehensive representation of weighted values, we referred each site’s weighted objectives to overall total weighted objectives for the chapter (N_Cit,W_), corresponding to relative frequencies (see above).

$$Relative\;Weighting\;(O;\;MFx)=\frac{Weighting\;(O;MFx)}{NCit,W\;(\Sigma MF;Chapt)}*100$$

For an overall view on the objectives, relative weightings of all faculties were summed up in a second step.

## Results

In the present study, the professional roles Collaborator and Manager (NKLM Chapts. 8 and 10) were analysed from a surgical point of view. The chapters were investigated separately; data relativization is based only on the corresponding chapter.

### Relative teaching frequency

The Mapping data reveal the current situation in surgical departments of eight medical faculties: in which fields of collaboration and management activity is found and how strong is its emphasis. The analysis focused on sub-competency level (Fig 1). The frequencies of the individual sub-competencies are shown in the context of the total overall number of citations for the corresponding chapter resulting in relative frequency values for the sub-competencies. The circle diameter encodes the number of faculties that explicitly teach a sub-competency.

The surgery departments of most faculties explicitly teach minor share of Collaborator’s sub-competencies (Fig 1A). However, for some sub-competencies higher citations in several faculties can be seen: Active and constructive participation in collaborative management of tasks (SC-8.1.1), appreciative behaviour in interprofessional collaboration (SC-8.2.1), development of a role identity as doctors (SC-8.3.1) and reflection on their function regarding continuous patient care (SC-8.3.2). These sub-competencies are taught either at four or more surgical departments or with a high frequency in single surgical departments. In contrast, the contents of other aspects of the role, summarized in competency C-8.4 (Solution of relevant healthcare issues in collaboration with other healthcare professions) are poorly represented. For the Manager, the following sub-competencies can be pointed out as they are taught either at four or more sites or with a high frequency in single sites (Fig 1B): Strategies for dealing with error and implementing patient safety (SC-10.6.1) and appropriate dealing with adverse events (SC-10.6.3). In contrast, the contents of competencies C-10.4 (allocation of resources), C-10.8 (use of time management), C-10.9 (career planning) and C-10.10 (development of leadership skills) are poorly represented.

Fig 1 shows that the complete spectrum of the Collaborator and the Manager is not explicitly covered in surgical curricula. Instead, particular aspects are emphasized: Collaboration within the medical team is more present than collaboration with other health professions concerning healthcare issues. Patient safety and handling of medical error and complication is clearly visible. Resource allocation, time management, career planning and leadership are poorly represented in surgical curricula.

Teaching of sub-competencies can result from teaching of one or more corresponding learning objectives. The distribution of these objectives is not always homogenous, as shown in Tables 1 and 2. It gives a more detailed overview of their relative frequencies and, moreover, for single faculties. Additionally, descriptive statistics for each objective are shown.

Complementary to Fig 1, Tables 1 and 2 show the distribution of mapping citations as relative frequencies over the single sites. They identify few sites to be responsible for high emphasis of single contents, whereas other sites give no or almost no contribution.

Highest mean values of learning objectives’ relative frequencies over all sites were reached for the objective O-8.1.1.3 (Reflection on respectful and appreciative treatment of team members, mean relative frequency 1.2) as well as involvement of relevant individuals and occupational groups in decision-making (O-8.1.1.1), contribution to team meetings (O-8.1.1.2), evaluation of team structures and processes targeting successful healthcare by interprofessional collaboration (O-8.2.1.1) and communication with the other healthcare professions (O-8.2.1.2, mean relative frequencies 1.1). These contents are all related to participation and behaviour within the medical team and, in parts, the interprofessional team. The findings on sub-competency level are thereby confirmed on objective level. Gaps in objectives of chapter 8 are restricted to competency C-8.4, as described above.

In chapter 10, highest mean values were reached for explaining factors influencing the development of complications (O-10.6.1.2, mean relative frequency 2.4) and application of error preventing strategies (O-10.6.1.4, mean relative frequency 1.7), widely according to the contents on sub-competency level. Gaps can be found in the competencies C-10.4, C-10.8, C-10.9 and C-10.10 as described above, but also in single objectives within almost all other competencies of the Manager. Only C-10.2 (familiarity with care structures), C-10.5 (models and methods of quality management) and C-10.6 (patient safety and immediate personal responsibility) are addressed, at least by one site, on learning objective level.

### Competency levels reached

For analysing the actual quality of performance, the highest competency levels achieved after five years of studies according to the mapping data were listed for each program in Fig 2. The site-specific specifications for the levels of objectives were compared to the NKLM requirements as given reference. The maximum competency level of the single sites teaching one learning objective is shown.

It is clear that the different sites teach at heterogeneous levels of competency. However, if an objective is taught in a surgical department at all, its competency level reaches or even exceeds the given NKLM reference. A striking difference between the Collaborator and Manager is the distinctly higher competency level achieved by a higher number of faculties for the objectives of the Collaborator.

Prominent objectives are found in competency C-8.1 (reflection on high quality of patient care and teamwork) and C-8.3 (work in medical team, contributing to effective care in the healthcare sector): involvement of relevant individuals and occupational groups in decision-making (O-8.1.1.1), contribution to team meetings (O-8.1.1.2), patient presentation and discussion of diagnostic and therapeutic procedures (O-8.3.1.2) as well as presentation of case histories (O-8.3.2.1). Obviously, quality and communication, especially in the medical team, are emphasized on that level, too. Objectives of the Collaborator, which are not included in surgical curricula, are restricted to competency C-8.4, as described above. The concerned objectives are: involve other professions in problem-solution processes (O-8.4.1.2), initiate joint processes for analysis and synthesis of problem-solving structures (O-8.4.1.3), use scientific findings and develop projects with other healthcare professions (O-8.4.2.1) and awareness as contributor to the healthcare sector besides other professions (O-8.4.2.2).

The prominent objectives of chapter 10 mainly belong to competency C-10.6, highlighted above, except of competency C-10.2: Identify involved care structures and plan treatments (O-10.2.1.1). The objectives distinguished from C-10.6 are: explaining factors influencing the development of complications (O-10.6.1.2), identify error types and mechanisms in concrete case examples (O-10.6.1.3), application of error preventing strategies (O-10.6.1.4) and recognition, reflection and addressing of own and others’ errors (O-10.6.3.1). As seen above, errors and complications are the most addressed topics of chapter 10.

### Relative weighting of learning objectives

For an overall assessment of the teaching intensity of each objective, the sum of the weighted frequencies (relative frequencies, multiplied by the corresponding competency levels) over all sites is shown in Fig 3. These relative weightings represent a ranking of learning objectives for the chapters 8 and 10, detached from their correlation to sub-competency and competency: The objectives that are taught most frequently and with highest levels of competency are plotted versus objectives that are not explicitly taught. For the Collaborator, most learning objectives of the chapter are taught; gaps were already described above (Fig 3A).

For the Manager, only 21 out of 37 objectives are addressed (Fig 3B), with few objectives reaching high relative weightings. The leading objectives are to reflect on respectful and appreciative treatment with team members (O-8.1.1.3) for the Collaborator and explaining factors influencing the development of complications (O-10.6.1.2) for the Manager. Both objectives also show the highest mean values for relative frequencies of their chapters (Tables 1 and 2).

Summary of findings, treatment of team members and involvement in decision making, as well as contribution in team meetings and presentation of patients represent the strengths of the Collaborator in surgical curricula. Although single objectives are strongly present, weaknesses can be found in interprofessional interaction, for example in problem solution, scientific work and in issues concerning the healthcare sector. The Manager has an emphasis on complication and error in surgical curricula. Gaps mainly concern structural, economic and personal issues. The frequency of the teaching content of the Collaborator and the Manager taking into account the competency levels shows a heterogeneous, but overall low integration in surgical curricula. At the most five of eight surgical departments teach specific contents explicitly, frequently and with a high competency level.

## Discussion

The current study questions whether the contents of the Collaborator and Manager are sufficiently taught in surgical curricula, including all obligatory surgical courses. On first sight, a low integration and heterogeneous distribution of these roles’ contents over the single sites becomes visible. A more detailed view shows that collaboration within the medical team is one of the higher represented topics aligning with existing research that was found [7–12]. However, in the current study collaboration is only partly covered as the important part of interprofessional cooperation, particularly in healthcare sector issues, is poorly represented. Patient safety and handling of errors and complications were found to be the most emphasized topics of the Manager. Besides collaboration, it represents one of the most important skills for a surgeon [9, 11]. However, Managers’ core competencies concerning structural, economic and personal topics like time management, career planning and leadership are not addressed. This shows that rather immediate and concrete skills are taught in surgery than superordinate skills. Interestingly, despite leadership, these superordinate skills were not explicitly estimated essential in the studies considered [10, 12]. Moreover, only very few curricula of the investigated faculties, including obligatory courses of all disciplines, address contents of the superordinate skills of the Manager at the recommended competency level 3b (competency in practice, independent). Thus, there seems to be a gap in teaching of these skills in undergraduate education.

Professional roles and the NKLM are in progress entering the medical curricula. In the current study we observed that several competencies, sub-competencies and objectives become more and more prominent in surgical education. However, it should be considered that only a few selected sites are responsible for high emphasis of single contents in this study, whereas some others give no or almost no contribution. These differences can in part be explained by the priorisation of certain topics by each faculty resulting in a local profile of peaks and gaps [26]. There are no specifications in the NKLM, which prove a single discipline to be responsible for the teaching of single sub-competencies and objectives. Therefore, from the perspective of a single discipline, priorisation can even have a stronger impact on profiles, especially gaps, as only a small number of courses are considered. This is particularly true for professional roles. However, these gaps and differences between faculties may stimulate outcome-driven discussion in surgical education. A direct comparison of the two chapters Collaborator and Manager is not possible due to the relativization on chapter level. However, it is obvious that content of the Collaborator is mediated more broadly and on higher competency levels in surgery compared to the Manager’s content. Due to this fact, different measures were applied for pointing out the most important objectives of the chapters. For the Manager, a stronger focus on specific topics becomes obvious. The question remains, whether these contents are really the most important for non-technical surgical education. Gaps in the citation of single objectives or sub-competencies are not relevant, as far as they do not concern surgical topics. But, are non-taught objectives sufficiently covered by other disciplines and are they really not necessary within a specific surgical context?

For single objectives of competencies concerning economic aspects of the healthcare system and leadership development, the NKLM gives no standards for undergraduate education. This means that learning of these contents is only recommended at a postgraduate stage. Moreover, these standards are given on the level of a curriculum, not a single discipline. Considering the curricula of the eight faculties involved in this study, professional roles are widely covered. However, specific contexts in surgery require special demands on these roles. As surgery, besides internal medicine, is one of the major disciplines, NKLM standards, as far as they concern professional roles, can be considered valid for surgery alone. The results show that, if an objective is taught in surgery at all, it reaches or even exceeds the NKLM standards. It indicates that surgical departments accept responsibility for teaching interdisciplinary roles, especially in context specific situations. Certainly, professional skills are further consolidated in obligate surgical courses and during residency. However, students still feel limited exposure to surgery during undergraduate education, leading to decisions against becoming a surgeon [27]. The German “Masterplan Medical studies 2020” to be passed, provides the implementation of practical education and the consolidation of teaching professional roles as well as interprofessional communication [28]. It is currently up for discussion among political and educational institutions in Germany [29].

Compared to explicit citations in the chapters 8 and 10 within the current study, implicit citations are clearly higher in all sites analysed. This also counts for the other professional roles of the NKLM. These findings were supported by Griewatz et al. who described discrepancies in favour of implicit compared to explicit teaching, particularly for the Collaborator in the German situation [30] and by Renting et al. who described the CanMEDS roles to occur in an integrated and usually implicit fashion [24]. An improvement of the awareness of professional roles could be reached by explicitly teaching the corresponding patterns of Manager and Collaborator in surgical courses. A Scottish surgical college, for example, runs popular regular courses that teach how to ensure safety through good communication and teamwork [6]. Moreover, several efforts have been described to adopt skills training from other disciplines like aviation: Davidson et al. describe many similarities, particularly in minimizing risk and managing complications. As they also state that safety developments in the operating room seem to have lagged behind other high risk organizations [31], there is need for action. In ideal circumstances, other professions are integrated in surgical training settings. Patient safety in surgery requires nontechnical skills such as teamwork. Indeed, there is prioritization of the improvement of nontechnical skills in surgery, which may be sustained by adopting teamwork training programs like Crew Resource Management (CRM) from other high-risk industries [32]. An integration of other relevant professions in such training scenarios can improve the outcome concerning competencies in interprofessional teams. As the results of the current study show, these can still be enhanced in surgical education. Lafleur et al. propose to use clinical studies to teach professional roles explicitly [33]. The method of curriculum mapping itself represents another possibility to sensitizes lecturers for objective based teaching, and effectively increased the number of taught objectives in surgery [34]. However, further attempts to support lecturers with the handling of the catalogue are necessary to achieve a competency-based curricular change [35].

### Limitations

The range in quantity of mandatory courses between the different surgical departments represents a limitation to the quantitative analysis. This range is due to the heterogeneous granularity of the local organizational structures (teaching units). Content-related overlaps of the chapters 8 and 10 with other NKLM chapters were not considered in the present study. It must be considered that the mapping data visualize a snapshot of surgical curricula which may develop continuously. Subjectivity of ratings cannot be excluded, despite of measures like consented procedures and plausibility checks. Although data cannot be taken as accurate values describing the curriculum precisely, they provide meaningful information, especially about the current status of professional roles in surgery.

## Conclusions

The study shows that overall involvement of surgery in teaching skills of the Collaborator and Manager is yet at a low level. However, single surgical departments commit themselves to explicitly teaching objectives of the Collaborator and Manager. This shows that the general acquisition of professional skills can be supplemented by the perspective of a single discipline. Specific demands on single roles can be considered as well as critical contexts for learning surgeon’s competencies. It is well known that implicitly taught professional roles are numerously present in surgical curricula, indicating awareness of professional roles. Addressing them explicitly is only a small step to go. Mapping data provide a reliable basis to decide whether the professional roles are adequately taught within discipline, faculty and cross-site.

## Supporting information

S1 Appendix NKLM chapters 8 and 10. (DOCX)
