# Supplementary material for: Surgeons’ participation in the development of collaboration and management competencies in undergraduate medical education
Source: PLoS One. 2020 Jun 5;15(6):e0233400. doi: 10.1371/journal.pone.0233400 (PMC7274374; doi:10.1371/journal.pone.0233400)
Supplement: S1 Appendix — (DOCX) [file pone.0233400.s001.docx]

# NKLM chapters 8 and 10

(unofficial translation)

| **ID** | **Content** |
| --- | --- |
| 8.1 | Graduates reflect on working together in a team and collaborate constructively in terms of high quality of patient care and teamwork. |
| 8.1.1 | They actively and constructively participate in teamwork in order to collaboratively manage tasks. They are able to… |
| 8.1.1.1 | Involve all relevant individuals and occupational groups in decision-making. |
| 8.1.1.2 | Actively and constructively contribute to team meetings. |
| 8.1.1.3 | Reflect on and, if necessary, change their own behaviour in terms of a respectful and appreciative treatment of the other team members. |
| 8.1.2 | They evaluate the quality of their joint work with the other team members and agree on improvement measures if required. They are able to… |
| 8.1.2.1 | Reflect on their own and others’ behaviour, recognize errors and address them in an appropriate way. |
| 8.1.2.2 | Develop a joint problem-solution strategy based on an error analysis and draw conclusions for their future joint work. |
| 8.1.2.3 | Constructively deal with self-criticism and third-party criticism and seek advice if necessary. |
| 8.2 | Graduates reflect on working together in a multi-professional team and collaborate constructively in terms of high quality patient care. |
| 8.2.1 | They show an appreciative behaviour in interprofessional collaboration, thereby contributing to good patient care. They are able to… |
| 8.2.1.1 | Evaluate team structures and processes against the background of the awareness that many fields in healthcare can only effectively succeed through interprofessional collaboration. |
| 8.2.1.2 | Communicate with the other healthcare professions in the appropriate technical language. |
| 8.2.1.3 | Integrate the perspectives and expert opinions of the involved occupational groups into their interprofessional collaboration and consider themselves to be part of a whole. |
| 8.2.2 | They possess a profession-specific role identity and know the roles, competencies and responsibilities of the other occupational groups involved. They are able to… |
| 8.2.2.1 | Explain their own tasks, areas of responsibility and limits within the interprofessional team in typical work situations and critically analyse their own actions in the overall process. |
| 8.2.2.2 | Actively deal with the roles and responsibilities of the other occupational groups and acknowledge their contribution to patient care. |
| 8.2.2.3 | Critically reflect on their own and other roles in experienced situations and, in the common communication process, draw conclusions on further steps to be taken. |
| 8.2.3 | They recognize interprofessional conflicts and actively contribute to productive and appropriate solutions. They are able to… |
| 8.2.3.1 | Analyse interprofessional conflicts and their origins and, in doing so, reflect on the different perspectives |
| 8.2.3.2 | Contribute to constructive solutions of interprofessional conflicts and participate in preventing them. |
| 8.3 | Graduates are qualified to work together in the medical team, thereby contributing to a high quality and effectiveness of care in the healthcare sector. |
| 8.3.1 | They develop a role identity as doctors. They are able to… |
| 8.3.1.1 | Explain their own tasks and responsibilities as well as their limits in collaborating with supervising and senior doctors. |
| 8.3.1.2 | Present patients, prioritize their problems and discuss the diagnostic and therapeutic procedures related to the individual patient and their situation in the team. |
| 8.3.2 | They reflect on their function with regard to continuous patient care. They are able to… |
| 8.3.2.1 | Present case histories in an appropriate, problem-related and clear manner in the medical record, during handover to colleagues on duty, in the findings report for service providers and in the doctor's letter. |
| 8.3.3 | They work together with various medical fields in an appropriate, appreciative and efficient manner. They are able to… |
| 8.3.3.1 | Explain the tasks and responsibilities of the general practitioner in medical care. |
| 8.3.3.2 | Explain the tasks and expertise of other medical fields as well as efficiently request the relevant consults. |
| 8.4 | In collaboration with other healthcare professions, graduates develop structures, processes and concepts which contribute to solving relevant healthcare issues. |
| 8.4.1 | They analyse existing healthcare structures in interaction with other healthcare professions and evaluate them with regard to their effectiveness and efficiency. They are able to… |
| 8.4.1.1 | Identify processes and structures which lead to ineffective and inefficient healthcare delivery processes in interaction with other healthcare professions and generate solutions for them. |
| 8.4.1.2 | Apply their healthcare knowledge and, in consultation with the other healthcare professions, involve further relevant individuals who support the implementation of problem-solving processes. |
| 8.4.1.3 | Initiate and moderate joint processes for the systematic analysis and synthesis of problem-solving structures. |
| 8.4.2 | Working together with other health professions, they develop concepts, guidelines, treatment paths etc. which support and continue the quality of care. They are able to... |
| 8.4.2.1 | Use scientific findings together with the other healthcare professions and develop projects for the scientific study of concepts with them. |
| 8.4.2.2 | See themselves and the other healthcare professions as contributors to the healthcare sector and consider the existing concepts and processes from a national and international perspective. |

| **ID** | **Content** |
| --- | --- |
| 10.1 | Graduates develop an understanding of their role as doctors. |
| 10.1.1 | They reflect on their own role as responsible persons and managers in the healthcare system. They are able to … |
| 10.1.1.1 | Name the laws, regulations and institutions governing the healthcare system and the social values (e.g. solidarity) underlying the healthcare system in Germany as well as describe and reflect on the role of medical doctors in this context. |
| 10.1.1.2 | Name and discuss the role and importance of doctors with regard to the change in structural framework conditions. |
| 10.1.1.3 | Name incentive structures, levels and forms and explain the effect of incentives. |
| 10.2 | Graduates are familiar with care structures. |
| 10.2.1 | They identify and analyse social care structures. They are able to … |
| 10.2.1.1 | Identify the involved care structures for a concrete patient and plan treatments with this knowledge in mind. |
| 10.3 | Graduates pay attention to the economic aspects of the healthcare system. |
| 10.3.1 | They describe the basic health economic structures and interrelations and are able to apply this knowledge to the concrete context of patient care and to participate in finding solutions to problems. They are able to… |
| 10.3.1.1 | Explain the basic economic framework conditions of the German healthcare system. |
| 10.3.1.2 | Present the basics of outpatient and inpatient remuneration systems and the importance of the coding with respect to remuneration/revenues. |
| 10.3.1.3 | Explain the procedure for the economic recognition of proven therapy methods. |
| 10.3.1.4 | Identify economic problems in the context of patient care and, in a dialogue, participate in finding solutions (i.e. distribution of resources). |
| 10.3.2 | They master efficient and effective medical work, they point out and define problems and work out solution approaches. They are able to… |
| 10.3.2.1 | Identify and separate medical, social, cultural, age- and gender-related issues in the case of concrete patients with acute helplessness, identify the economic conflict, if required, and help to prepare a decision. |
| 10.3.2.2 | Map a concrete care situation to the DRG system and assess the consequences of their medical decisions with regard to billing. |
| 10.4 | Graduates handle the allocation of resources in a responsible manner. |
| 10.4.1 | They identify patient care situations which require decisions regarding the distribution of resources and participate in the decision-making process. They are able to… |
| 10.4.1.1 | Take and justify a position regarding the decisions of “(un)fitness for work”, “occupational disability” and “partial or full, temporary or permanent reduction in earning capacity” and their sociomedical consequences using their knowledge of the possibilities for rehabilitation on the basis of medical and legal criteria in a given medical patient situation. |
| 10.4.1.2 | Take and justify a position regarding the decision to apply for recognition of the “degree of disability” and possible sociomedical consequences based on medical and legal criteria in a given medical patient situation. |
| 10.4.1.3 | Take and justify a position regarding the decision to apply for “levels of care” in a given medical patient situation. |
| 10.4.2 | They are familiar with the methods of resource allocation (on different levels and for different providers of services). They are able to… |
| 10.4.2.1 | Understand and explain instruments of resource allocation in practice as well as resulting conflicts. |
| 10.5 | Graduates know the models and methods of quality management and apply them. |
| 10.5.1 | They are aware of quality assurance measures in patient care and their areas of application. They are able to… |
| 10.5.1.1 | Name and participate in measures of quality assurance in a specific disease situation. |
| 10.6 | Graduates pay attention to patient safety and are aware of their immediate personal responsibility. |
| 10.6.1 | They address key aspects of dealing with errors and use strategies for implementing patient safety. They are able to… |
| 10.6.1.1 | Define and differentiate critical events, damages and errors (system failures) as well as terms such as "error", "complication" or "adverse event" and identify them in a specific case. |
| 10.6.1.2 | Explain different factors influencing the development of complications. |
| 10.6.1.3 | Name different types of errors, theoretically explain mechanisms by which errors occur and identify them in a concrete case example. |
| 10.6.1.4 | Apply error-prevention strategies. |
| 10.6.1.5 | Create an event report based on a given medical context with a critical event and differentiate possible consequences. |
| 10.6.2 | They know key aspects of complication management, risk communication and the Critical Incident Reporting System (CIRS). They recognize critical events and are trained in dealing with wrong decisions. They are able to… |
| 10.6.2.1 | Explain the importance of a safety culture for reporting critical events and learning from them. |
| 10.6.2.2 | Identify and classify occurred complications as well as name medical, organisational and communicative consequences for damage containment/prevention. |
| 10.6.3 | They demonstrate that they can deal with adverse events appropriately. They are able to… |
| 10.6.3.1 | Reflect on their own behaviour and that of others, recognize errors and address them appropriately to their colleagues and supervisors. |
| 10.6.3.2 | Adequately communicate errors to patients and their families. |
| 10.6.3.3 | Perform an adequate, systematic error analysis on themselves and others. |
| 10.7 | Graduates use rational decision-making. |
| 10.7.1 | They use information technology (IT) to procure and transfer information and to document treatment processes. They are able to… |
| 10.7.1.1 | Explain and apply the tasks, function and components of a hospital information system (HIS). |
| 10.7.1.2 | Request examinations in the Clinical Workplace System, document findings, as well as create a medication prescription and a doctor's letter. |
| 10.7.1.3 | Apply information systems for outpatient care as well as explain the doctor’s responsibilities in operating these systems. |
| 10.7.1.4 | Apply procedures for the secure transmission and storage of patient data and assess whether patient data has been sufficiently anonymized so that it may be processed outside of the treatment context. |
| 10.7.1.5 | Use telemedicine solutions in a patient-oriented manner and explain framework conditions for health telematics. |
| 10.8 | Graduates use time management. |
| 10.8.1 | They develop strategies for setting priorities and effective time management. They are able to… |
| 10.8.1.1 | Develop a work schedule for a given day and prioritize it using the example of inpatient care. |
| 10.9 | Graduates work on their career planning. |
| 10.9.1 | They develop career goals when starting their career planning, taking into account and communicating work-life balance when implementing these goals. They are able to… |
| 10.9.1.1 | Point out and communicate the possibilities and limits of professional (further training, leading positions) and academic (doctorate, habilitation) career planning on the basis of concrete data. |
| 10.9.2 | They plan their own careers and identify their personal qualification needs. They are able to… |
| 10.9.2.1 | Compile and present the possibilities and conditions (and funding, if applicable) of the specific further training programme and/or academic qualification. |
| 10.10 | Graduates develop leadership skills. |
| 10.10.1 | They deal with their role as a young team members and their future development of a leadership personality. They are able to… |
| 10.10.1.1 | Review the documented decision-making process according to hierarchical, occupational group-related, communication-based and medicolegal  aspects as well as present and critically assess the roles of those involved based on a given case history. |
| 10.10.1.2 | Perceive, reflect on and adequately address patients’ and team members’ expectations of medical management responsibility. |
| 10.10.2 | They know different leadership styles and assume management functions. They are able to… |
| 10.10.2.1 | Identify different leadership styles and management tasks in learning situations and working structures and reflect on their effectiveness. |
| 10.10.2.2 | Assume management responsibilities in a team in concrete situations (e.g. learning and study groups, working groups, ward team) |
